# Central venous catheter-related complications in older haemodialysis patients: A multicentre observational cohort study

**DOI:** 10.1177/11297298221085225

**Published:** 2022-03-31

**Authors:** Mathijs van Oevelen, Boudewijn DC Heggen, Alferso C Abrahams, Joris I Rotmans, Maarten GJ Snoeijs, Robin WM Vernooij, Marjolijn van Buren, Sabine CA Meijvis

**Affiliations:** 1 Department of Internal Medicine, Leiden University Medical Center, Leiden, The Netherlands; 2 Department of Vascular Surgery, Maastricht University Medical Center, Maastricht, The Netherlands; 3 Department of Nephrology and Hypertension, University Medical Center Utrecht, Utrecht, The Netherlands; 4 Julius Center for Health Sciences and Primary Care, University Medical Center Utrecht, Utrecht University, Utrecht, The Netherlands; 5 Department of Nephrology, Haga Hospital, The Hague, The Netherlands

**Keywords:** Haemodialysis, vascular access, catheter, complication, infection, elderly

## Abstract

**Background:**

Central venous catheters (CVC) remain a commonly used vascular access option in haemodialysis, despite guidelines advising to preferably use arteriovenous fistulae. Compared to younger patients, the risk-benefit ratio of CVC in older patients might be more beneficial, but previous studies mainly focussed on catheter-related bacteraemia and/or assessed tunnelled CVC (TCVC) only. This study’s aim was to compare all catheter-related infections and malfunctions in older patients with younger patients using all CVC subtypes.

**Materials and methods:**

We used data from DUCATHO, a multicentre observational cohort study in The Netherlands. All adult patients in whom a CVC was placed for haemodialysis between 2012 and 2016 were included. The primary endpoint was the occurrence of catheter-related infections, comparing patients aged ⩾70 years with patients aged <70 years (reference). As secondary endpoints, catheter malfunctions and catheter removal due to either infection or malfunction were assessed. Using Cox proportional hazards and recurrent events modelling, hazard ratios (HR) with 95% confidence intervals (CI) were calculated with adjustment of prespecified confounders. Additionally, endpoints were assessed for non-tunnelled CVC (NTCVC) and TCVC separately.

**Results:**

A total of 1595 patients with 2731 CVC (66.5% NTCVC, 33.1% TCVC) were included. Of these patients, 1001 (62.8%) were aged <70 years and 594 (37.2%) ⩾70 years. No statistically significant difference was found for the occurrence of catheter-related infections (adjusted HR 0.80–95% CI 0.62–1.02), catheter malfunction (adjusted HR 0.94–95% CI 0.75–1.17) and catheter removal due to infection or malfunction (adjusted HR 0.94–95% CI 0.80–1.11). Results were comparable when assessing NTCVC and TCVC separately.

**Conclusion:**

Patients aged ⩾70 to <70 years have a comparable risk for the occurrence of catheter-related infections and catheter malfunction. These findings may help when discussing treatment options with older patients starting haemodialysis and may inform the current debate on the best vascular access for these patients.

## Introduction

For patients on maintenance haemodialysis, a safe and reliable vascular access is vital. Guidelines advise to create autologous arteriovenous fistulae (AVF) as the preferred option and resort to central venous catheters (CVC) only when no other vascular access options are available.^1–3^ CVC are associated with a up to five times higher risk of vascular access-related infections, in particular bacteraemia (i.e. catheter-related bloodstream infection, CRBSI), and up to three times higher risk of vascular access malfunction, compared to AVF.^4^ AVF, however, pose several disadvantages too: they require suitable vessels, sufficient time for maturation and even then up to one in three AVF fail to mature.^5,6^ In older patients, the risk-benefit ratio of CVC versus AVF might differ from younger patients. Due to ageing and more severe vascular pathology, suitable vessels for fistula creation might be lacking and the risk of maturation failure is even higher.^7,8^ Additionally, the required surgical procedure and its perioperative process pose an increased risk for morbidity in older patients.^9^ Furthermore, the added benefit of AVF might be more limited in older patients as the supposed long-term benefit might not be reached. In a large study in the United States, over 30% of predialysis patients aged 70–75 years who underwent AVF or arteriovenous graft (AVG) surgery either died before dialysis initiation or did not require dialysis within 2 years and this proportion further increased with age.^10^ After dialysis is initiated, mortality is significantly higher in older patients: in the Netherlands, 18% of patients ⩾70 years die within their first year of treatment, compared to 8% for patients <70 years (Personal communications of M.O. with Nefrovisie Foundation, based on data from 2019 to 2020 from the Dutch Renal Replacement Registry (RENINE)). Daily practice shows that in elderly patients, CVC are still an often chosen option: nearly 80% of patients in the United States aged ⩾75 years start dialysis using a CVC and after a year nearly 30% of these patients remains CVC-dependent.^11^

Due to several mechanisms, including increasing comorbidities and deteriorating immune responsiveness as age increases, older patients in general are often regarded as more prone to complications, including infections and thrombosis.^12,13^ Our hypothesis was that older patients also have more catheter-related complications, including catheter-related infections, compared to younger patients. Prior studies that compared catheter-related complications in older patients had conflicting results^14^ and/or assessed CRBSI only (Table 1).^14–19^ Moreover, most of these studies restricted their inclusion to tunnelled CVC (TCVC) only.^14,16–20^ Although our previous study showed that precurved non-tunnelled CVC (NTCVC) are comparable to TCVC in terms of infections and catheter malfunction, it is unknown whether this premise holds true for the older population.^21^ Hence, the primary aim for this study was to compare the occurrence of catheter-related infections between patients ⩾70 years to patients <70 years using both NTCVC and TCVC. Secondly, we assessed the occurrence of catheter malfunctions (e.g. catheter-related thrombosis or material problems) and the removal of the catheter due to either catheter-related infection or malfunction for older versus younger patients.

**Table 1.** Previous studies on catheter-related infections and/or malfunction.

Authors	Country (centres)	Period	Patients	CVC	Type of CVC	Outcome measure(s)	Comp	Ref	Outcome (95% CI)
Zanoni et al.^15^	Italy (1)	2014–2017	413	560	39% TCVC	CRBSI	⩾80	18–79	HR 0.66 (0.30–1.47)^a^
					61% NTCVC				HR 0.60 (0.28–1.27)^b^
Mohamed et al.^14^	Ireland (1)	2015–2016	139	NS	100% TCVC	CRBSI	⩾75	<75	RR 1.42 (0.79–2.45)^c^
									RR 1.38 (0.78–2.45)^d^
Poinen et al.^20^	Canada (5)	2004–2012	1041	First only	100% TCVC	CVC-related complications within 2 years	60 to 69	<60	HR 0.83 (0.67–1.03)^e^
							70 to 79	<60	**HR 0.67 (**0.52–0.85**)**^e^
							⩾80	<60	**HR 0.69 (**0.52–0.92**)**^e^
Yap et al.^16^	Singapore (1)	2010–2012	677	First only	100% TCVC	CRBSI	⩾65	<65	OR 1.36 (0.88–2.10)^c^
Murea et al.^17^	USA (9)	2005–2007	464	NS	100% TCVC	CRBSI	⩾75	18–74	**HR 0.34 (**0.21–0.55**)**^c^
									**HR 0.33 (**0.20–0.55**)**^f^
Shingarev et al.^22^	USA (1)	2004–2009	472	First only	100% TCVC	CVC removal due to infection/dysfunction	⩾65	<65	HR 1.01–0.73–1.40)^c^
						CRBSI			HR 0.91 (0.65–1.28)^c^
Lemaire et al.^18^	France (4)	1982–2005	1749	2230	100% TCVC	CRBSI	⩾50.3 to <63.6	<50.3	OR 0.67 (0.44–1.01)^c^
							⩾63.6 to <72.6	<50.3	OR 0.67 (0.44–1.01)^c^
							⩾72.6	<50.3	OR 0.89 (0.60–1.33)^c^
Marr et al.^19^	USA (4)	1995–1996	102	NS	100% TCVC	CRBSI	⩾55	<55	RR 0.97 (0.61–1.56)^c^

Only studies comparing age groups, reporting ORs, HRs or RRs, and with a minimum of 100 patients are shown. Statistically significant outcomes highlighted in bold.

CI: confidence interval; comp: comparator group; CRBSI: catheter-related bloodstream infection; CVC: central venous catheter(s); HR: hazard ratio; NS: not specified; (N)T: (non)-tunnelled; OR: odds ratio; ref: reference group; RR: relative risk/risk ratio.

a Adjusted for CVC type (NTCVC vs TCVC), sex and acute kidney injury.

b Adjusted for insertion site (femoral vs jugular), sex and acute kidney injury.

c Unadjusted.

d Adjusted for insertion site, sex and diabetes.

e Adjusted for sex, BMI, diabetes, history of cancer, cardiovascular disease and inpatient dialysis start.

f Adjusted for ancestry, dialysis vintage, diabetes mellitus, coronary artery disease, congestive heart failure, dialysis unit, initial TCVC site, first catheter lock solution and immunosuppression.

## Materials and methods

### Study design and population

This study used data from the *DUtch CATHeter Outcomes* (DUCATHO) study.^21^ The DUCATHO study was a retrospective multicentre cohort study in 12 Dutch centres which included patients between January 1st, 2012 and December 31st, 2016. The study was approved by the Medical Research Ethics Committee of the University Medical Center Utrecht. Obtaining informed consent was waived since data were collected and processed anonymously. All adult patients in whom a CVC was placed for haemodialysis during this period were included, including subsequent CVC in individual patients. CVC were excluded if they were used for continuous venovenous hemofiltration in the intensive care unit, if patients objected to use their medical record for research purposes or if patients underwent haemodialysis in a non-participating centre during the study period. Follow-up was recorded using electronic patient files and conducted from placement to catheter removal, death or end of study period (December 31st, 2016). For this study, patients with missing age were excluded from analyses.

### Outcomes and definitions

#### Primary and secondary endpoints

The primary endpoint of the study was the occurrence rate of catheter-related infections. The two secondary endpoints consisted of (i) the occurrence rate of catheter malfunction and (ii) the removal of the CVC due to either a catheter-related infection or catheter malfunction. For each CVC, a maximum of four events (either infection or catheter malfunction) was consecutively recorded in detail. Any following events were only registered as either being present or not, without further details.

#### Catheter-related infections

These infections included all exit site infections, tunnel infections and systemic infections. Exit site infections were diagnosed if erythema, induration and/or pain near the insertion site of the CVC were present in combination with positive cultures from secretions. Tunnel infections were diagnosed if tenderness, induration and/or erythema of the skin and subcutaneous tissue were present along the insertion site and tunnelled route of the CVC, in combination with positive cultures from secretions. Systemic infections were defined as the presence of positive blood cultures, general clinical symptoms of infection, such as fever or raised inflammatory parameters, and when the CVC was deemed the most likely source of infection by the treating physician. Patients were also considered as having a systemic infection when they had clinical signs of infection, without any other likely source apart from the CVC, and when the infection was treated as a CRBSI with systemic antibiotics. When multiple subtypes of infection were present within the same episode (e.g. a tunnel infection leading to CRBSI) the most severe subtype (i.e. systemic infections first, followed by tunnel- and exit site infections) was scored.

#### Catheter malfunction

Catheter malfunction was defined as absent or low haemodialysis blood flows that impaired effective haemodialysis delivery and required treatment, as judged by the treating physician. This included thrombosis, catheter material problems or dysfunction due to other causes. Thrombosis was defined as a formed thrombus which attaches to the inner or outer surface of the catheter. Catheter material problems were defined as when catheters tore or hubs were dysfunctional. Potential treatments for catheter malfunction included use of thrombolytics such as urokinase, CVC guidewire exchange, radiologic intervention, catheter removal or surgical intervention. When multiple subtypes malfunction were present within the same episode (e.g. a material problem leading to thrombosis) the most severe subtype (i.e. thrombosis first, followed by material problems and unknown/other malfunctions) was scored.

### Statistical analyses

For this study, patients were stratified into two age groups: patients of 70 years or older and patients younger than 70 years old. For all outcomes, cause-specific hazard ratios (HR) were calculated using patients aged ⩾70 years as comparison and <70 years as reference. For each patient, all CVC that were inserted during the study period were used for main analyses. Additionally, NTCVC and TCVC were assessed separately. To account for recurrent events for the analyses of infection and/or catheter malfunction events, a Prentice, Williams and Peterson model was used as subsequent events are not independent.^23^ For the secondary endpoint ‘catheter removal due to either infection or catheter malfunction’, a Cox proportional hazards model was used. The proportional hazards assumption was verified using Schoenfeld residuals. All results are shown as unadjusted (crude) and adjusted HRs: on theoretical grounds and based on the original DUCATHO study, sex, history of diabetes mellitus, cerebrovascular or peripheral vascular disease, use of relevant medication (i.e. immunosuppressive drugs for endpoints involving infections, antiplatelet drugs and anticoagulants for endpoints involving catheter malfunction) and subtype of CVC (i.e. straight NTCVC, precurved NTCVC or TCVC) were identified as potential confounders and entered in the model.

Descriptive statistics were expressed as counts (*n*) and percentages (%). Continuous data were expressed as means with standard deviations (SD) for normally distributed data and medians with interquartile ranges (IQR) for non-normally distributed data. A *p*-value of <0.05 was considered statistically significant. Descriptive statistics and univariable analyses were performed using SPSS (version 20.0). Nominal data was compared using chi-squared-tests, continuous data using *t*-tests for normally distributed data. Multivariable analyses were performed in R Studio (version 3.5.1).

### Sensitivity analyses

To resemble a chronic haemodialysis population in varying extends, we performed three sensitivity analyses: we (i) excluded CVC used for short-term dialysis (defined as a maximum of two dialysis sessions performed, e.g. for intoxications or acute kidney failure), (ii) excluded CVC inserted in femoral position, as these are likely to have a higher complication risk and are generally not used for maintenance haemodialysis and (iii) restricted analyses to CVC used for more than 90 days.

## Results

The original DUCATHO study enrolled 1603 patients with 2746 CVC. For the following analyses, an additional eight patients (0.5%) were excluded due to missing age, resulting in 1595 patients with 2731 CVC with a total of 268,582 catheter days (Table 2). Mean age at placement of the first CVC was 62.4 years, 594 (37.2%) were 70 years or older and 1001 (62.8%) were less than 70 years old. Older patients more often had comorbidities (i.e. diabetes mellitus, cerebrovascular disease and peripheral arterial disease) and more commonly used anticoagulant or antiplatelet drugs. Younger patients more often used immunosuppressive drugs, their dialysis more frequently had an acute start, and less frequently was categorised as short-term.

**Table 2.** Baseline characteristics of all enrolled patients (left) and stratified by age (mid/right).

	Total (*n* = 1595)	⩾70 years (*n* = 594)	<70 years (*n* = 1001)	*p*-Value*
Age at placement of first CVC (years)	62.4 ± 15.7	77.3 ± 5.1	53.6 ± 12.9	<0.001
Male sex	936 (58.7)	333 (56.1)	603 (60.2)	0.101
BMI (kg/m^2^)	26.3 ± 5.4	26.1 ± 4.9	26.4 ± 5.7	0.214
Comorbidity				
Diabetes mellitus	581 (36.4)	242 (40.7)	339 (33.9)	0.006
Peripheral artery disease	209 (13.1)	95 (16.0)	114 (11.4)	0.009
Cerebrovascular disease	236 (14.8)	121 (20.4)	115 (11.5)	<0.001
Medication				
Anticoagulants	634 (39.7)	272 (45.8)	362 (36.2)	<0.001
Antiplatelet drugs	506 (31.7)	231 (38.9)	275 (27.5)	<0.001
Immunosuppression	438 (27.5)	119 (20.0)	319 (31.9)	<0.001
Dialysis characteristics				
Acute start	636 (39.9)	204 (34.3)	432 (43.2)	0.001
Short-term^†^	130 (8.2)	61 (10.3)	69 (6.9)	0.018

CVC: central venous catheter; BMI: body mass index; SD: standard deviation.

Data presented as mean ± SD or count (%).

* Comparison of groups ⩾70 to <70 years old.

† Defined as less than three dialysis sessions.

Of the used CVC, 1815 (66.5%) were non-tunnelled and 904 (33.1%) were tunnelled – the remaining 12 CVC (0.4%) had no registered subtype (Table 3). NTCVC were in situ much shorter than TCVC (median 23 vs 115 days). Of the NTCVC, 796 (43.9%) were precurved, 975 (53.7%) were straight and 44 (2.4%) were of unknown subtype.

**Table 3.** Characteristics of used CVC, stratified by age group and CVC subtype.

	⩾70 years			<70 years		
	all CVC (n = 1019)	NTCVC (*n* = 690)	TCVC (*n* = 325)	all CVC (*n* = 1712)	NTCVC (*n* = 1125)	TCVC (*n* = 579)
Catheter days (median, IQR)	39 [10 – 120]	21 [8 – 78]	113 [46–261]	43 [12 − 128]	25 [9 − 79]	117 [39 –233]
Total number of catheter days	99,408	38,076	61,221	169,174	68,734	100,283
Subtype						
Straight	–	310 (44.9)	–	–	486 (43.2)	–
Precurved	–	367 (53.2)	–	–	608 (54.0)	–
Unknown	–	13 (1.9)	–	–	31 (2.8)	–
Length of catheter						
<20 cm	459 (45.0)	453 (65.7)	5 (1.5)	757 (44.2)	750 (66.7)	7 (1.2)
20–24 cm	260 (25.5)	171 (24.8)	89 (27.4)	410 (23.9)	243 (21.6)	167 (28.8)
25 cm or more	214 (21.0)	32 (4.6)	182 (56.0)	425 (24.8)	92 (8.2)	332 (57.3)
Unknown	86 (8.4)	34 (4.9)	49 (15.1)	120 (7.0)	40 (3.6)	73 (12.6)
Diameter of catheter						
<12 French	110 (10.8)	96 (13.9)	14 (4.3)	336 (19.6)	318 (28.3)	18 (3.1)
12–13.5 French	311 (30.5)	277 (40.1)	34 (10.5)	481 (28.1)	379 (33.7)	101 (17.4)
14 French or more	489 (48.0)	238 (34.5)	251 (77.2)	759 (44.3)	339 (30.1)	418 (72.2)
Unknown	109 (10.7)	79 (11.4)	26 (8.0)	136 (7.9)	89 (7.9)	42 (7.3)
Location of placement						
Jugular	784 (76.9)	496 (71.9)	287 (88.3)	1345 (78.6)	851 (75.6)	492 (85.0)
Subclavian	37 (3.6)	24 (3.5)	13 (4.0)	51 (3.0)	34 (3.0)	15 (2.6)
Femoral	185 (18.2)	162 (23.5)	20 (6.2)	286 (16.7)	222 (19.7)	62 (10.7)
Other or unknown	13 (1.3)	8 (1.2)	5 (1.5)	30 (1.8)	18 (1.6)	10 (1.7)
Complications						
Had one or more catheter-related infection	187 (18.4)	110 (15.9)	77 (23.7)	362 (21.1)	174 (15.5)	188 (32.5)
Had one or more catheter malfunction	319 (31.3)	185 (26.8)	133 (40.9)	628 (36.7)	370 (32.9)	254 (43.9)
Had more than four complications	29 (2.8)	8 (1.2)	21 (6.5)	59 (3.4)	27 (2.4)	31 (5.4)
Catheter-related infections (per 1000 catheter days)	2.35	3.55	1.62	2.70	3.22	2.35
Exit site	1.09	1.81	0.64	1.22	1.73	0.87
Tunnel	0.06	–	0.10	0.10	–	0.17
Systemic	1.21	1.73	0.88	1.35	1.41	1.31
Catheter malfunction events (per 1000 catheter days)	5.55	7.75	4.18	6.20	8.39	4.64
Thrombosis	1.09	1.60	0.77	1.54	2.08	0.29
Material problem	0.45	0.71	0.29	0.41	0.58	0.29
Other or unknown	4.01	5.44	3.12	4.25	5.73	4.06
Reason for removal						
Catheter-related infection	123 (12.1)	73 (10.6)	50 (15.4)	191 (11.2)	96 (8.5)	95 (16.4)
Catheter malfunction	129 (12.7)	84 (12.2)	44 (13.5)	270 (15.8)	165 (14.7)	102 (17.6)
End of dialysis treatment or use of AVF/AVG	422 (41.4)	302 (43.8)	119 (36.6)	822 (48.0)	571 (50.8)	249 (43.0)

AVF: arteriovenous fistula; AVG: arteriovenous graft; CVC: central venous catheter; IQR: interquartile range; (N)T: (non)-tunnelled.

Data presented as count (%) unless stated otherwise. Numbers might not add up to 100% due to missing values and rounding.

### Catheter-related complications

Using all CVC, 18.4% of all patients ⩾70 years old had one or more infectious complications and 31.3% had one or more catheter malfunction event (Table 3 and Supplemental Table S1). For patients aged <70 years, these were 21.1%–36.7%, respectively. The catheter-related infection rate was 2.35 per 1000 catheter days for patients ⩾70 years and 2.70 for patients <70 years old. For catheter malfunctions this was 5.55–6.20 per 1000 catheter days, respectively. NTCVC had higher complication rates than TCVC. For patients aged ⩾70 years, 12.1% of CVC were removed due to catheter-related infections and 12.7% due to catheter malfunctions. For patients <70 years old, this was 11.2% for catheter-related infections and 15.8% for catheter malfunctions.

### Primary and secondary endpoints

All endpoints are shown in Table 4. For the unadjusted primary endpoint (i.e. occurrence of catheter-related infection), no statistically significant association was found for patients aged ⩾70 years compared to patients <70 years (HR 0.90–95% CI 0.71–1.14). Both unadjusted secondary endpoints (i.e. occurrence of catheter malfunction and removal due to catheter-related infection or malfunction) also showed no statistically significant association (HR 0.94–95% CI 0.75–1.16 and HR 0.95–95% CI 0.81–1.12 respectively). When NTCVC and TCVC were assessed separately, results were comparable to those for all CVC combined. After adjustment for prespecified confounders (i.e. sex, history of diabetes mellitus, cerebrovascular disease and peripheral arterial disease, subtype of CVC when assessing all CVC, use of immunosuppressive drugs for endpoints involving infection and use of antiplatelet drugs or anticoagulants in endpoints involving catheter malfunction), again no statistically significant association was found: HR 0.80 (95% CI 0.62–1.02) for the primary endpoint, HR 0.94 (95% CI 0.75–1.17) for the occurrence of catheter malfunction and HR 0.94 (95% CI 0.80–1.11) for CVC removal.

**Table 4.** Multivariable analyses.

	Unadjusted HR (95% CI)	*p*-Value	Adjusted HR* (95% CI)	*p*-Value
Primary endpoint: occurrence of catheter-related infection				
⩾70 years vs <70 years (reference), all CVC	0.90 (0.71–1.14)	0.38	0.80 (0.62–1.02)	0.08
⩾70 years vs <70 years (reference), NTCVC only	0.95 (0.71–1.28)	0.75	0.89 (0.66–1.20)	0.44
⩾70 years vs <70 years (reference), TCVC only	0.69 (0.46–1.04)	0.08	0.68 (0.43–1.07)	0.09
Secondary endpoint: occurrence of catheter malfunction				
⩾70 years vs <70 years (reference), all CVC	0.94 (0.75–1.16)	0.55	0.94 (0.75–1.17)	0.57
⩾70 years vs <70 years (reference), NTCVC only	1.00 (0.78–1.28)	1.00	1.00 (0.77–1.30)	0.99
⩾70 years vs <70 years (reference), TCVC only	0.83 (0.56–1.25)	0.38	0.81 (0.54–1.21)	0.31
Secondary endpoint: removal due to catheter-related infection or malfunction				
⩾70 years vs <70 years (reference), all CVC	0.95 (0.81–1.12)	0.56	0.94 (0.80–1.11)	0.48
⩾70 years vs <70 years (reference), NTCVC only	1.03 (0.85–1.25)	0.75	1.03 (0.84–1.26)	0.78
⩾70 years vs <70 years (reference), TCVC only	0.80 (0.59–1.07)	0.13	0.80 (0.59–1.09)	0.16

CI: confidence interval; CVC: central venous catheter; HR: hazard ratio; (N)T: (non)-tunnelled.

* Adjusted for age, sex, history of diabetes, cerebrovascular disease and peripheral vascular disease (all analyses), type of CVC (i.e. precurved NTCVC, straight NTCVC or TCVC, excluding analyses with TCVC/NTCVC only), use of immunosuppressive drugs (primary endpoint and removal due to infection/malfunction only) and use of antiplatelet drugs or anticoagulants (secondary endpoints only).

### Sensitivity analyses

When analyses were repeated excluding CVC used for short-term dialysis, CVC placed in femoral position, or patients with less than 90 days of dialysis, results were comparable: no statistically significant difference between patients ⩾70 to <70 years was found for all endpoints (Table 5).

**Table 5.** Multivariable analysis – sensitivity analyses.

	Unadjusted HR (95% CI)	*p*-Value	Adjusted HR* (95% CI)	*p*-Value
Primary endpoint: occurrence of catheter-related infection				
⩾70 years vs <70 years (reference), excluding short-term dialysis^†^	0.88 (0.69–1.12)	0.31	0.78 (0.60–1.00)	0.05
⩾70 years vs <70 years (reference), excluding femoral CVC	0.88 (0.68–1.13)	0.30	0.82 (0.63–1.06)	0.13
⩾70 years vs <70 years (reference), chronic dialysis only^‡^	0.77 (0.53–1.12)	0.17	0.82 (0.56–1.19)	0.29
Secondary endpoint: occurrence of catheter malfunction				
⩾70 years vs <70 years (reference), excluding short-term dialysis^†^	0.92 (0.73–1.14)	0.44	0.92 (0.73–1.15)	0.46
⩾70 years vs <70 years (reference), excluding femoral CVC	0.99 (0.78–1.26)	0.95	1.02 (0.80–1.31)	0.85
⩾70 years vs <70 years (reference), chronic dialysis only^‡^	0.94 (0.62–1.41)	0.75	0.86 (0.58–1.28)	0.46
Secondary endpoint: removal due to catheter-related infection or malfunction				
⩾70 years vs <70 years (reference), excluding short-term dialysis^†^	0.93 (0.79–1.10)	0.43	0.92 (0.78–1.09)	0.34
⩾70 years vs <70 years (reference), excluding femoral CVC	0.96 (0.80–1.15)	0.64	0.98 (0.82–1.18)	0.85
⩾70 years vs <70 years (reference), chronic dialysis only^‡^	0.78 (0.58–1.07)	0.12	0.84 (0.63–1.11)	0.22

CI: confidence interval; CVC: central venous catheter; HR: hazard ratio; (N)T: (non)-tunnelled.

* Adjusted for age, sex, history of diabetes, cerebrovascular disease and peripheral vascular disease, type of CVC (i.e. precurved NTCVC, straight NTCVC or TCVC), use of immunosuppressive drugs (primary endpoint and removal due to infection/malfunction only) and use of antiplatelet drugs or anticoagulants (secondary endpoints only).

† Defined as less than three dialysis sessions.

‡ Defined as more than 90 days of dialysis.

## Discussion

Our study shows a comparable risk for patients aged ⩾70 to <70 years for the occurrence of either catheter-related infections or malfunctions. Also, when assessing the removal of CVC due to either infection or malfunction, no significant differences were found. These results were similar when assessing NTCVC and TCVC separately. Although no statistically significant difference between both age groups was found, there was a numerically lower risk for all endpoints in patients aged ⩾70 years. Most notably, a 20% reduction of the occurrence of infectious events (up to 32% when only including TCVC), would pose a clinically relevant difference for older compared to younger patients.

Our results are in line with several previous studies. Poinen et al.^20^ found a lower risk (HR 0.67–95% CI 0.52–0.85) for Canadian patients aged 70–79 years compar-ed to those <60 years for catheter-related complications. Complications in this study included all CVC-related procedures, hospitalisations and death within 2 years of placement of a TCVC. Our point estimates for the occurrence of either catheter-related infections or malfunctions using TCVC only (HR 0.68–95% CI 0.43–1.07 and HR 0.81–95% CI 0.54–1.21, respectively) were very comparable. This agreement is notably interesting as the use of CVC in prevalent dialysis patients in Canada is exceptionally common while in the Netherlands this is more rare (approximately 45% vs 12% in 2013) (Personal communications of M.O. with Nefrovisie Foundation, based on data for 2013 from the Dutch Renal Replacement Registry (RENINE)).^24^ Regional differences should be taken into account when assessing the risks and benefits of various vascular access options, as the population at risk likely differs significantly due to patient selection.

Murea et al. found an even lower risk for older American patients with TCVC (HR 0.33–95% CI 0.20–0.55) using an age cut-off of 75 years, but restricted their outcome to CRBSI.^17^ Although CRBSI is an important, potentially life-threatening complication, other CVC-related infections and malfunctions can also lead to significant morbidity and/or loss of the CVC and these were included in our study. Zanoni et al.^15^ also assessed CRBSI only but included both NTCVC and TCVC, using a cut-off of 80 years. The authors, like our study, found no statistically significant difference with a trend towards lower risk in older patients (HR 0.66–95% CI 0.30–1.47). Multiple other studies also did not show any association for older patients and varying catheter-related outcomes (Table 1).^14,16,18,19,22^

The observed trend towards a lower complication risk in older patients in our and aforementioned studies is remarkable, as older patients are often regarded as more prone for infectious and thrombotic complications in general. Multiple hypotheses have been proposed as to why older patients would have fewer CVC-related complications. Due to more limited physical activity, less manipulation of the CVC will occur, possibly limiting its exposure to pathogens. Similarly, others suggested decreased transpiration seen in older patients as a possible mechanism as one study found higher rates of catheter-related septicaemia with higher ambient temperatures.^17,25,26^ Finally, one study showed less frequent nasal *Staphylococcal aureus* colonisation in older patients and subsequently showed an increased risk for bacteraemia in carriers.^27^

Traditionally, CVC are seen as more complication-prone than AVF. Studies comparing CVC with either AVF have largely been biased, for example by only using AVF that were successfully created instead of also including failed AVF. Confounding is also an important factor in these mainly observational studies: as AVF are seen as superior, mostly patients with disadvantageous characteristics (e.g. acute start of dialysis, unfavourable vasculature, more severe comorbidity or limited life-expectancy) are started and often remain on CVC. The new KDOQI guidelines acknowledge this and have nuanced the traditional ‘fistula first, catheter last’ advise to ‘*right access, in the right patient, at the right time, for the right reasons’*.^3^ Here patients’ goals and preferences, life expectancy and comorbidities are more taken into account. Nevertheless, the proportion of patients using AVF or CVC remains an often-used key performance indicator. Even though our study does not compare the complication risks of CVC with AVF, its results do emphasise that, in elderly patients, perhaps CVC are not as complication prone as traditionally thought, particularly TCVC.

Our study has several distinctive strengths. First, our study uses the largest cohort to date on CVC-related outcomes in older patients (Table 1). This allows us to reliably assess outcomes. We used three clinically relevant endpoints and did not limit our scope to CRBSI only. As mentioned, less severe infections (e.g. tunnel infections) or catheter malfunctions can also lead to loss of vascular access and/or increased morbidity. Second, we both assessed all CVC combined and TCVC and NTCVC separately: previous studies only included TCVC,^14,16–20,22,27,28^ only described TCVC and NTCVC combined^15,29,30^ or even included all types of vascular access.^31,32^ Our study adds that age has no effect on outcomes in both subtypes of CVC. Finally, the use of multiple sensitivity analyses strengthens the assumption that the observed results are applicable to both short-term- and chronic haemodialysis patients.

Our study also has several limitations. First, a retrospective design is generally considered prone to misclassification bias. However, by using clear, predefined definitions for the outcomes of interest, this effect was limited. Second, we had no available data on catheter-related mortality or hospitalisations. Finally, only a maximum of four events for each CVC were registered in DUCATHO and CVC with previous complications are likely more at risk for new complications. As a result, the reported incidence rates are an underestimation. However, as the number of CVC with more than five complications was low (3.2%), this effect will be limited.

The main clinical implication of our study is emphasising the critical evaluation of all vascular access options, including CVC, in older patients instead of simply extending the *‘fistula first’* approach to this complex patient population. Our study shows that older patients have a CVC-associated complication risk that is at least comparable to younger patients. Although a null effect could not be ruled out, a 32% lower risk for infections in older patients using TCVC compared to younger patients would clinically be relevant. In these older patients, perhaps a (T)CVC should more frequently be discussed as a suitable vascular access option. Besides complication risks, other factors should be taken into account in the decision-making process. For example, a study on patients’ preferences showed that older patients with CVC reported comparable satisfaction and less access-related symptoms compared to those with AVF.^33^ With the ever ageing dialysis population, more patients with complex comorbidities and/or limited life expectancy will require a well-informed and shared decision on what vascular access option is most suitable for them. Well-designed studies are needed to directly compare AVF, AVG and CVC in these older patients. As confounding by indication is an important limitation for observational studies comparing these vascular access types, a randomised-controlled design is preferred. In the Netherlands, the *Optimising Access Surgery In Senior* *haemodialysis* *patients* (OASIS) trial, comparing AVF, AVG and CVC, is currently ongoing using a randomised-controlled design.^34^

To summarise, our study showed that patients aged 70 years or older have a comparable risk for catheter-related infections and malfunction compared to younger patients. These findings may help when discussing treatment options with older patients starting haemodialysis and may inform the current debate on the best vascular access for these patients.

## Supplemental Material

sj-docx-1-jva-10.1177_11297298221085225 – Supplemental material for Central venous catheter-related complications in older haemodialysis patients: A multicentre observational cohort study Click here for additional data file. Supplemental material, sj-docx-1-jva-10.1177_11297298221085225 for Central venous catheter-related complications in older haemodialysis patients: A multicentre observational cohort study by Mathijs van Oevelen, Boudewijn DC Heggen, Alferso C Abrahams, Joris I Rotmans, Maarten GJ Snoeijs, Robin WM Vernooij, Marjolijn van Buren and Sabine CA Meijvis in The Journal of Vascular Access
