# Supplementary material for: Central venous catheter-related complications in older haemodialysis patients: A multicentre observational cohort study
Source: J Vasc Access. 2022 Mar 31;24(6):1322–31. doi: 10.1177/11297298221085225 (PMC10714686; doi:10.1177/11297298221085225)
Supplement: sj-docx-1-jva-10.1177_11297298221085225 – Supplemental material for Central venous catheter-related complications in older haemodialysis patients: A multicentre observational cohort study [file sj-docx-1-jva-10.1177_11297298221085225.docx]

**SUPPLEMENTAL MATERIAL**

| **Table S1. Absolute number of events per CVC, stratified by age groups** | | |
| --- | --- | --- |
|  | **Catheter-related**  **infection** | **Catheter**  **malfunction** |
| **1 event** |  |  |
| ≥70 years | 155/1019 (15.2) | 186/1019 (18.3) |
| <70 years | 297/1712 (17.3) | 376/1712 (22.0) |
| **2 events** |  |  |
| ≥70 years | 21/1019 (2.1) | 66/1019 (6.5) |
| <70 years | 42/1712 (2.5) | 137/1712 (8.0) |
| **3 events** |  |  |
| ≥70 years | 7/1019 (0.7) | 34/1019 (3.3) |
| <70 years | 16/1712 (0.9) | 61/1712 (3.6) |
| **4 events** |  |  |
| ≥70 years | 4/1019 (0.4) | 33/1019 (3.2) |
| <70 years | 7/1712 (0.4) | 54/1712 (3.2) |
| **Any number of events** |  |  |
| ≥70 years | 187/1019 (18.4) | 319/1019 (31.3) |
| <70 years | 362/1712 (21.1) | 628/1712 (36.7) |
| Data shown as count (%). A maximum of 4 events per included CVC was recorded. | | |
